# Application of serum proteomics to the Women's Health Initiative conjugated equine estrogens trial reveals a multitude of effects relevant to clinical findings

**DOI:** 10.1186/gm47

**Published:** 2009-04-29

**Authors:** Hiroyuki Katayama, Sophie Paczesny, Ross Prentice, Aaron Aragaki, Vitor M Faca, Sharon J Pitteri, Qing Zhang, Hong Wang, Melissa Silva, Jacob Kennedy, Jacques Rossouw, Rebecca Jackson, Judith Hsia, Rowan Chlebowski, JoAnn Manson, Samir Hanash

**Affiliations:** 1 Molecular Diagnostics Program, Fred Hutchinson Cancer Research Center, Fairview Avenue North, Seattle, WA 98109, USA; 2 Laboratory of Core Technology, Eisai Co. Ltd, 5-1-3 Tokodai, Tsukuba, Ibaraki 300-2635, Japan; 3 Department of Pediatrics, University of Michigan, Cancer Center, 1500 E. Medical Center Drive, Ann Arbor, MI 48109, USA; 4 Public Health Sciences Division, Fred Hutchinson Cancer Research Center, Fairview Avenue North, Seattle, WA 98109, USA; 5 Women's Health Initiative Branch, National Heart, Lung, and Blood Institute, Rockledge Dr., Bethesda, MD 20892, USA; 6 Division of Endocrinology, Ohio State University, Dodd Dr., Columbus, OH 43210, USA; 7 AstraZeneca LP, Concord Pike, Wilmington, DE 29850, USA; 8 Los Angeles Biomedical Research Institute at Harbor-UCLA Medical Center, W. Carson Street, Torrance, CA 90502, USA; 9 Brigham and Women's Hospital, Harvard Medical School, Boylston Street, Boston, MA 02215, USA

## Abstract

**Background:**

The availability of serum collections from the Women's Health Initiative (WHI) conjugated equine estrogens (CEE) randomized controlled trial provides an opportunity to test the potential of in-depth quantitative proteomics to uncover changes in the serum proteome related to CEE and to assess their relevance to trial findings, including elevations in the risk of stroke and venous thromboembolism and a reduction in fractures.

**Methods:**

Five independent large scale quantitative proteomics analyses were performed, each comparing a set of pooled serum samples collected from 10 subjects, 1 year following initiation of CEE at 0.625 mg/d, relative to their baseline pool. A subset of proteins that exhibited increased levels with CEE by quantitative proteomics was selected for validation studies.

**Results:**

Of 611 proteins quantified based on differential stable isotope labeling, the levels of 116 (19%) were changed after 1 year of CEE (nominal *P *< 0.05), while 64 of these had estimated false discovery rates <0.05. Most of the changed proteins were not previously known to be affected by CEE and had relevance to processes that included coagulation, metabolism, osteogenesis, inflammation, and blood pressure maintenance. To validate quantitative proteomic data, 14 proteins were selected for ELISA. Findings for ten - IGF1, IGFBP4, IGFBP1, IGFBP2, F10, AHSG, GC, CP, MMP2, and PROZ - were confirmed in the initial set of 50 subjects and further validated in an independent set of 50 additional subjects who received CEE.

**Conclusions:**

CEE affected a substantial fraction of the serum proteome, including proteins with relevance to findings from the WHI CEE trial related to cardiovascular disease and fracture.

**Clinical Trials Registration:**

ClinicalTrials.gov identifier: NCT00000611

## Background

Estrogens exert effects on target genes in various tissues through complex processes [1]. Given the widespread use of conjugated equine estrogens (CEE) and other estrogens for menopausal symptoms, the issue of overall health benefits and risks associated with CEE has been a major research focus. For example, recommendations for use of estrogen for prevention of coronary heart disease (CHD) were based on epidemiologic, animal, and laboratory data [2,3]. However, the Women's Health Initiative (WHI) randomized, placebo controlled trial of 0.625 mg/d continuous CEE among 10,739 women who were post-hysterectomy did not provide evidence of benefit for CHD, and health benefits and risks appeared to be approximately balanced [4]. It has been suggested that women who started CEE earlier after menopause could be at lower risk of CHD, but not stroke, than women who initiated hormone therapy more distant from the menopause [5-8]. Demonstrated benefits of CEE include improvement of vasomotor symptoms [9] and prevention of osteoporotic fractures, in particular reduction in hip fractures [10,11]. Adverse effects observed in the WHI trial include increased incidence of venous thromboembolism and stroke [4,12,13].

Recent studies, including the WHI trials, have shown that estrogen therapy (ET) induced changes in several proteins and metabolites, including decreases in low-density lipoprotein cholesterol and increases in high-density lipoprotein cholesterol and triglycerides; decreases in fasting glucose, insulin, and homocysteine; increases in C-reactive protein, matrix metalloproteinase-9 and plasmin-antiplasmin complex; and decreases in E-selectin and plasmin activator inhibitor [14]. Other studies have documented increases in angiotensinogen and its product angiotensin II, a potent vasoconstrictor, and suppression of active renin with postmenopausal ET [15,16]. There is also some evidence of an effect on insulin-like growth factor (IGF) and IGF binding proteins (IGFBPs) in postmenopausal women [17,18]. Given these diverse effects, an unbiased comprehensive profiling of serum to assess the effect of CEE is warranted. However, such comprehensive quantitative proteomic profiling in the context of a clinical trial has not been done previously. Thus, it was of interest to determine whether proteomic profiling would uncover protein changes that have relevance to WHI CEE trial findings.

We have applied an intact protein analysis system (IPAS) approach that allows identification of proteins over seven orders of magnitude of abundance to determine the effect of oral CEE on the serum proteome [19-22]. A prior proteomic study of hormone therapy-relevant samples [23] relied on a fingerprinting approach with limited sensitivity and without protein identification. In this study we present a systematic global proteome analysis of sera obtained at baseline and after 1 year of oral ET from 50 postmenopausal women. We have validated quantitative proteomic data for a subset of proteins by enzyme-linked immunosorbent assay (ELISA) with sera from the initial set of 50 subjects and with sera from an independent set of 50 randomly selected subjects who adhered to CEE and that were obtained at baseline and after 1 year of oral ET.

## Methods

### Study design

Use of human samples was approved by the Fred Hutchinson Cancer Research Center Institutional Review Board. For the discovery phase of this study, 50 subjects were randomly selected from women in the WHI trial who received and adhered to oral CEE 0.625 mg daily over the first year from randomization, and who did not experience a major clinical outcome during trial follow-up. This population is a substudy of the WHI CEE trial, which is composed of 10,739 women, 5,310 in the active CEE arm and 5,429 in the placebo arm. These women had each undergone hysterectomy, and most had never received hormone therapy prior to trial enrollment. Some were prior postmenopausal hormone therapy users who had stopped hormone therapy some months or years prior to trial enrollment. Rarely, subjects were current hormone therapy users at baseline screening and these subjects were required to undergo a 3 month 'wash-out' period of no hormone therapy use prior to randomization. Sera were collected before and after 1 year of CEE in 7 ml royal blue-stoppered serum tubes for trace elements, no additive, silicone coated (BD 367737), and frozen at -80°C until proteomic analysis. All subjects in this substudy were adherent to study medication (defined as taking >80% of study medication per protocol) throughout the first year from randomization. Sera from a second subgroup (n = 50) of women from the active CEE arm of the CEE trial who met the same selection criteria were included in an independent sample ELISA validation phase of this study.

### Sample preparation

Sera samples at baseline and 1 year after ET (50 women total) were divided in 5 experiments. For each experiment 30 μl aliquots of sera from 10 women at baseline, and 10 women 1 year after ET were pooled. Baseline and treated pools were then individually immunodepleted of the top six most abundant proteins (albumin, IgG, IgA, transferrin, haptoglobin and antitrypsin) using a Hu-6 column (4.6 × 250 mm; Agilent, Wilmington, DE, USA). Briefly, columns were equilibrated with buffer A at 0.5 ml/minutes for 13 minutes and aliquots of 75 μl of the pooled sera were injected after filtration through a 0.22 μm syringe filter. The flow-through fractions were collected for 10 minutes at a flow rate of buffer A of 0.5 ml/minute, combined and stored at -80°C until use. The column bound material was recovered by elution for 8 minutes with buffer B at 1 ml/minute. Subsequently, immunodepleted samples were concentrated using Centricon YM-3 devices (Millipore, Billerica, MA, USA) and re-diluted in 8 M urea, 30 mM Tris pH 8.5, 0.5% OG (octyl-beta-d-glucopyranoside; Roche Diagnostics, Indianapolis, IN, USA). Samples were reduced with DTT in 50 μl of 2 M Tris-HCl pH 8.5 (0.66 mg DTT/mg protein), and isotopic labeling of intact proteins in cysteine residues were performed with acrylamide. Baseline pools received the light acrylamide isotope (C12 acrylamide; >99.5% purity; Sigma-Aldrich (Fluka), St. Louis, MO, USA), and their corresponding 1 year ET pools received the heavy 1,2,3-C13-acrylamide isotope (C13 acrylamide; >98% purity; Cambridge Isotope Laboratories, Andover, MA, USA). Alkylation with acrylamide was performed for 1 h at room temperature by adding to the protein solution the appropriate quantity of C12-acrylamide or C13-acrylamide per milligram protein, diluted in a small volume of 2 M Tris-HCl pH 8.5 [19]. For each of the five experiments, the pool of baseline (C12) and estrogen-treated (C13) samples was then mixed together for further analysis.

### Protein fractionation

The two-dimensional protein fractionation has been performed based on the previously described IPAS approach [20,22,24]. Briefly, after isotopic labeling and mixing of the two pools, the sample was diluted to 10 ml with 20 mM Tris in 6% isopropanol, 4 M urea pH 8.5 and immediately injected in a Mono-Q 10/100 column (Amersham Biosciences, Piscataway, NJ, USA) for the anion-exchange chromatography, the first dimension of the protein fractionation. The buffer system consisted of solvent A (20 mM Tris in 6% isopropanol, 4 M urea pH 8.5) and solvent B (20 mM Tris in 6% isopropanol, 4 M urea, 1 M NaCl pH 8.5). The separation was performed at 4.0 ml/minutes in a gradient of 0-35% solvent B in 44 minutes; 35-50% solvent B in 3 minutes; 50-100% solvent B in 5 minutes; and 100% solvent B for an additional 5 minutes. A total of 12 pools were collected from the anion exchange chromatography. The 12 pools were then subjected to a second dimension of separation by reversed-phase chromatography. The reversed-phase fractionation was carried out with a Poros R2 column (4.6 × 50 mm; Applied Biosystems, Foster City, CA, USA) using trifluoro-acetic acid/acetonitrile as buffer system (solvent A, 95% H_2_O, 5% acetonitrile, 0.1% trifluoro-acetic acid; solvent B, 90% acetonitrile, 10% H_2_O, 0.1% trifluoro-acetic acid) at 2.7 ml/minutes. The gradient used was 5% solvent A until absorbance reached baseline (desalting step) and then 5-50% solvent B in 18 minutes; 50-80% solvent B in 7 minutes and 80-95% solvent B in 2 minutes. Sixty fractions of 900 μl were collected during the run, corresponding to a total of 720 fractions for each experiment. Aliquots of 200 μl of each fraction, corresponding to approximately 20 μg of protein, were separated for mass-spectrometry shotgun analysis.

### Mass spectrometry analysis

For protein identification we performed in-solution trypsin digestion with the lyophilized aliquots of the 720 individual fractions. Individual digested fractions 4 to 60 from each reversed-phase run were pooled in 11 pools, corresponding to a total of 132 fractions for analysis from each experiment. Tryptic peptides were analyzed by a LTQ-FT mass spectrometer (Thermo-Electron, Waltham, MA USA) coupled to a nano-Aquity nanoflow chromatography system (Waters, Milford, MA, USA). The liquid chromatography separation was performed in a 25 cm column (Picofrit 75 μm ID; New Objective, Woburn, MA, USA), in-house-packed with MagicC18 (Michrom Bioresources, Auburn, CA, USA) resin using a 90 minutes linear gradient from 5-40% of acetonitrile in 0.1% formic acid at 250 nl/minute. The spectra were acquired in a data-dependent mode in a m/z range of 400 to 1,800, and selection of the 5 most abundant +2 or +3 ions of each mass spectrometry (MS) spectrum for MS/MS analysis. Mass spectrometer parameters were: capillary voltage of 2.1 KV; capillary temperature of 200°C; resolution of 100,000; and FT target value of 1,000,000.

### Protein identification

The acquired LC-MS/MS data were automatically processed by the Computational Proteomics Analysis System (CPAS) [25]. For the identification of proteins with a false discovery rate (FDR) <5%, database searches were performed using X!Tandem against the human IPI (International Protein Index) database v.3.13 using tryptic search [25]. Cysteine alkylation with the light form of acrylamide was set as a fixed modification and with the heavy form of acrylamide (+3.01884) as a variable modification. The database search results were then analyzed by PeptideProphet [26] and ProteinProphet [27] programs. Our high confidence list of identifications retained proteins with ProteinProphet scores ≥0.95 (5% error rate) and two or more peptides per protein.

### Quantitative analysis of protein levels

Quantitative ratios of proteins comparing 1-year to baseline samples were obtained by differential labeling of peptides containing cysteine with acrylamide isotopes (heavy or light). Quantitative information was extracted using a script designated 'Q3ProteinRatioParser' that was developed in-house to obtain the relative quantification for each pair of peptides identified by MS/MS that contains cysteine residues [19]. Only peptides with a minimum PeptideProphet score of 0.75, and mass deviation <20 ppm were considered for quantification. Ratios of heavy-to-light acrylamide-labeled peptides were plotted on a histogram (log2 scale) and the median of the distribution was centered at zero. This normalization approach was chosen since the great majority of proteins were not expected to be deregulated in 1-year ET compared to baseline samples. All normalized peptide ratios for a specific protein were averaged to compute an overall protein ratio. Proteins for which only peptides labeled with the heavy form of acrylamide were detected were included in the final list of proteins with quantitative information presented as '1-year ET only'. All peptide and protein ratios were calculated on a logarithmic scale. Statistical significance of the protein quantitative information was obtained via two procedures: for those proteins with multiple peptides quantified, a *P*-value for the mean log-ratio, which has mean zero under the null hypothesis, was calculated using one-sample *t*-test; and for proteins with a single paired MS event, the probability for the ratio was extrapolated from the distribution of ratios in a baseline-baseline experiment whereby the same sample was labeled with heavy and light acrylamide. The raw data and summary list of identified and quantified proteins are available through the Computational Proteomics Analysis System upon request.

### Statistical comparison of five IPAS proteomics analyses

Protein ratios were analyzed to identify proteins whose average ratio (1 year of CEE/baseline), averaged over the five proteomic experiments, differed from zero on a log2 scale. All analyses were performed using the statistical package R [28]. Protein log-ratios were normalized across experiments by a median location shift to ensure the distributions of proteins for each IPAS experiment were centered at zero. Protein log-ratios were standardized by forming a sample variance from the (up to five) log-ratios for each protein, and adding a corresponding sample variance from a corresponding set of (up to five) log-ratios from a completely analogous set of five proteomic experiments from the WHI estrogen plus progestin trial. Statistical testing was performed by using a weighted moderated t-statistic [29] implemented in the R package LIMMA [30]. A weighted average ratio was calculated for each protein by weighting the (up to five) log-ratios by the number of quantified peptides for each protein and a matrix of weights was included in the linear model. Benjamini and Hochberg's method for controlling the FDR was used to compute adjusted *P*-values [31].

To improve our estimate of the posterior standard deviation used in the moderated t-statistics, protein ratios from an additional five IPAS experiments that compare estrogen plus progestin and whose quantification followed exactly the same protocol were also included in the linear model. Specifically, average ratios were calculated by fitting a linear model where the design matrix consisted of two dummy variables indicating estrogen or estrogen plus progestin use. All results in this manuscript are based on inferences for the dummy variable of estrogen use (that is, the average ratio for ET use). Including the estrogen plus progestin data does not affect the estimated values of the ET ratios, but does increase the degrees of freedom and consequently increases power.

### Networks analysis

For network analysis, the unfiltered list of gene names of proteins, and their ratios and *P*-values from all five IPAS experiments were uploaded into the MetaCore analytical suite version 4.7 (GeneGO, Inc., St. Joseph, MI, USA), and analysis was performed as described previously [32].

### ELISA-based validation

Measurements were performed on the same sera from the 50 women utilized for proteomic analysis using ELISAs according to the manufacturer's protocols: human IGFBP1, IGFBP2, IGFBP4, and IGFBP6 (R&D Systems, Minneapolis, MN, USA); IGF1 (Diagnostic Systems Laboratories, Webster, TX, USA); factor IX (F9), factor X (F10), and PROZ (protein Z, vitamin K-dependent plasma glycoprotein) (Hyphen Biomed, Neuville-Sur-Oise, France); ceruloplasmin (US Biological, Swampscott, MA, USA); vitamin D binding protein (Alpco Diagnostics, Salem, NH, USA); fetuin-A (AHSG) (Biovendor, Candler, NC, USA); vitronectin (Innovative Research, Novi, MI, USA); KNG1 (Affinity Biologicals, Ancaster, ON, Canada); MMP2 (Calbiochem, Gibbstown, NJ, USA). Individual serum samples and standards were run in duplicate and absorbance measured using a SpectraMax Plus 384 and results calculated with SoftMax Pro v4.7.1 (Molecular Devices, Sunnyvale, CA, USA). *P*-values and testing whether there was a significant change from baseline to year 1 for individual proteins were computed using the non-parametric *t*-test on the log2 scale. For a particular protein, validity of IPAS results was gauged by comparing means (95% confidence intervals) of protein ratios to results from standard ELISA kits. The t-statistic and moderated t-statistic were used to calculate 95% confidence intervals for ELISA and IPAS data. For comparison of discovery and validation findings we also report Pearson's correlation coefficients for log-ratios.

## Results

### Proteomic analysis of sera from study subjects

Some characteristics at baseline of the 50 subjects included in the discovery phase are summarized in Table 1. There were no statistically significant differences in any baseline characteristics noted between pools. The average age of the subjects was 61.4 ± 7.9 years (mean ± standard deviation).

**Table 1:** Overview of subject characteristics (n = 50)

	N	%
Age group at screening, years		
50-59	25	50.0
60-69	13	26.0
70-79	12	24.0
Ethnicity		
White	42	84.0
Black	5	10.0
Hispanic	3	6.0
Hormone replacement therapy use		
Never used	26	52.0
Past user	19	38.0
Current user	5	10.0
Hormone replacement therapy duration, years		
<5	16	66.7
5 to <10	3	12.5
10+	5	20.8
Body mass index (BMI), kg/m^2^		
<25	6	12.2
25 to <30	18	36.7
≥30	25	51.0
BMI at year 1		
<25	3	6.1
25 to <30	21	42.9
≥30	25	51.0
Smoking		
Never smoked	29	58.0
Past smoker	19	38.0
Current smoker	2	4.0
Parity		
Never pregnant/no term pregnancy	4	8.0
≥1 term pregnancy	46	92.0
Age at first birth, years		
<20	15	34.1
20-29	27	61.4
30+	2	4.5
Age at hysterectomy, years		
<40	19	38.0
40-49	18	36.0
50-54	6	12.0
55+	7	14.0
Prior bilateral oophorectomy		
No	33	71.7
Yes	13	28.3
Treated diabetes		
No	43	86.0
Yes	7	14.0
Treated for hypertension or blood pressure ≥140/90 mmHg		
No	29	63.0
Yes	17	37.0
History of high cholesterol requiring pills		
No	42	95.5
Yes	2	4.5
Statin use at baseline		
No	48	96.0
Yes	2	4.0
Aspirin (≥80 mg) use at baseline		
No	42	84.0
Yes	8	16.0
History of myocardial infarction		
No	50	100.0
History of angina		
No	47	94.0
Yes	3	6.0
History of coronary artery bypass graft/percutaneous transluminal coronary angioplasty		
No	47	100.0
History of stroke		
No	50	100.0
History of deep vein thrombosis or pulmonary embolism		
No	50	100.0
Family history of breast cancer (female)		
No	41	85.4
Yes	7	14.6
History of fracture on/after age 55		
No	31	96.9
Yes	1	3.1
Gail Model five year risk of breast cancer		
<1	10	20.0
1 to <2	34	68.0
2 to <5	6	12.0
Number of falls in last 12 months		
None	30	69.8
1	7	16.3
2	6	14.0

**Figure 1:**
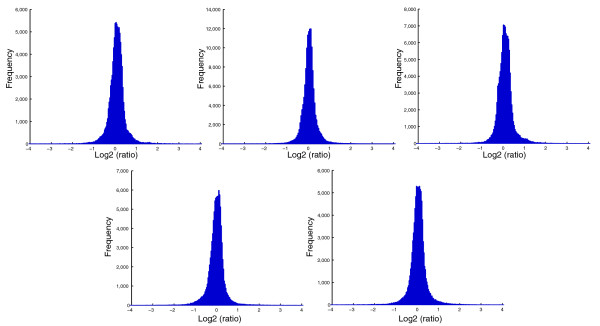
Distribution of ratios for quantified peptides for the five IPAS experiments. A histogram of 1-year CEE/baseline (log2) ratios as determined from heavy-to-light isotopic labeling with acrylamide are shown for each IPAS experiment. The median of the distribution was centered at zero for normalization.

There were 2,576,869 tandem mass spectra with >0.05 PeptideProphet score acquired in these experiments (Table 2); 1,760,094 spectra yielded proteins identified with a <5% error rate. To our knowledge, this serum protein dataset is the largest obtained from a human observational study or clinical trial to date. This remarkable size of the serum protein dataset is a result of the extensive fractionation and large number of mass spectra collected in these experiments. The number of proteins identified and quantified showed some variation between experiments (16% coefficient of variation for number of quantified proteins), which may be related to sample processing and MS sampling. However, this variation is not expected to affect quantitative ratios, as each experiment consisted of combined baseline and post-therapy sera that were differentially isotopically labeled prior to mixing. Labeling efficiency was evaluated and the results are shown in Figure 1. The log-ratio histograms were all approximately Gaussian shaped.

**Table 2:** Overview of proteomic analysis characteristics

Experiment	Number of tandem mass spectra acquired	Number of spectra that yielded protein identifications with <5% error rate	Number of unique proteins quantified
1	414,895	293,466	543
2	584,525	403,189	574
3	524,366	355,411	575
4	573,327	381,057	530
5	479,756	326,971	370
			
Total	2,576,869	1,760,094	1,056

### Changes observed at 1 year following ET relative to baseline

**Figure 2:**
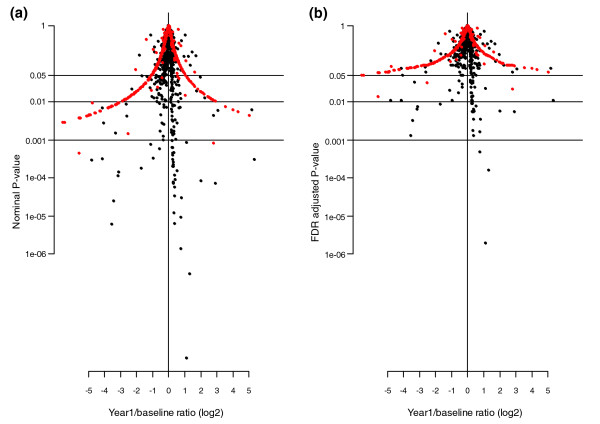
Volcano plots. **(a) **For nominal *P*-values. Relationship between the 1-year ET/baseline log2 ratios and their *P*-values. **(b) **For FDR adjusted *P*-values. Relationship between the 1 year ET/baseline log2 ratios and their FDR adjusted *P*-values.

A list of weighted, quantified protein products of 611 distinct genes resulted from the serum proteomic analysis (Additional data file 1), after filtering protein identifications to remove proteins without associated gene name (hypothetical proteins) and false identifications based on manual verification of mass spectra. The log2 ratios of protein levels (1 year CEE/baseline), derived from the isotopic labeling of cysteine residues, and their *P*-values is provided as volcano plots (Figure 2a). We found that 116 of the 611 proteins quantified in the serum met a nominal 0.05 significance level criterion for change after 1 year of CEE, compared to about 31 expected by chance. A similar view was obtained when adjusted *P*-values (FDR <0.05) were considered (Figure 2b). We found that 64 of the 611 proteins quantified (10.5%) in the serum had estimated FDRs of *P *< 0.05 for change from baseline to 1 year from randomization (Additional data file 2), while a strongly overlapping set of 64 proteins had nominal *P *< 0.05 and also had estimated log-ratios >1.20 or <1/1.20 (Additional data file 3). A network analysis of the 64 proteins with statistically significant changes relative to all quantified proteins and with an FDR <0.05 (MetaCore version 4.7) [32-35] yielded a significant enrichment in five networks: blood coagulation, kallikrein-kinin system, cell adhesion-platelet-endothelium-leukocyte interactions, complement system, and ossification (Table 3). We further classified these 64 proteins in relation to the known biological processes they are involved in through a search of the Gene Ontology (GO) database (Table 4). A search of the literature yielded prior associations with ET for 13 of the 64 proteins (ceruloplasmin (CP), plasminogen (PLG), tissue factor pathway inhibitor (TFPI), sex hormone binding globulin (SHBG), IGFBP1, IBFBP4, apolipoprotein A-II (APOA2), vitamin D binding protein (GC), apolipoprotein D (APOD), IGF1, AHSG, lactotransferrin (LTF), angiotensinogen (AGT); Table 4). Thus, novel associations were observed for 41 proteins. These proteins are associated primarily with blood coagulation, metabolism regulation, complement/inflammation/innate immunity, ossification, cellular growth, cell-cell/cell-matrix interactions, vessel morphogenesis/angiogenesis and blood pressure maintenance processes.

**Table 3:** Significant GeneGo biological networks for proteins that met a FDR <0.05

Network number	Name	*P*-value	Network objects	Objects in the network*
1	Blood coagulation	1.66 e-6	7/83	UP: F12, F9, F10, PROZ, SERPING1, MST1
				DOWN: MMP2
2	Complement system	1.57 e-4	5/73	UP: SERPING1, C2 (C2a, C2b)
				DOWN: MBL2
3	Kallikrein-kinin system	2.84 e-4	7/183	UP: PLG, SERPING1, F9, F10, F12, HABP2
4	Cell adhesion, cell matrix interactions	6.34 e-4	7/209	UP: VTN, TGFBI, HABP2, LGALS3BP, LGALS1
				DOWN: MMP2, COL1A1
5	Platelet-endothelium-	1.42 e-3	6/175	UP: PLG, F12, F10, SERPING1, VTN
	leukocyte interactions			DOWN: MMP2
6	Ossification	4.34 e-3	5/152	UP: INHBE, IGFBP4, IGFBP1-IGFBP6
				DOWN: IGF1, TLL1
7	Cell proliferation	5.4 e-3	5/160	UP: IGFBP1, IGFBP4, IGFBP6
				DOWN: IGF1, MMP2
8	Protein C signaling	6.05 e-3	4/103	UP: PLG, F9, F10
				DOWN: EDG3

*UP and DOWN refer to up-regulated and down-regulated, respectively. C2, complement c2; LGALS3BP, galectin-3-binding protein; MBL2, mannose-binding protein C; NOTCH, neurogenic locus notch homolog protein 2; TGFBI, transforming growth factor-beta-induced protein ig-h3.

**Table 4:** Classification of proteins with statistically significant changes based on Gene Ontology

Protein	Log2 ratio year one relative to baseline	P-value
**Blood coagulation and inflammation**		
Vitronectin (VTN)	0.374	9.27E-08
Ceruloplasmin (CP) [59]	0.789	1.51E-06
Plasminogen (PLG) [51]	0.307	1.50E-06
Kininogen (KNG1) [52]	0.265	2.89E-05
Coagulation factor XII (F12) [52]	0.364	4.64E-05
Coagulation factor IX (F9)	0.558	8.34E-05
Coagulation factor X (F10)	0.332	0.00029
Carboxypeptidase N, polypeptide 1 (CPN1)	0.288	0.0002
Platelet basic protein (PPBP)	0.273	0.00363
Tissue factor pathway inhibitor (TFPI) [51]	-0.267	0.01152
Fibrinogen gamma chain (FGG)	0.273	0.01848
Matrix metalloproteinase 2 (MMP2)	-0.681	0.03019
Protein Z, vitamin K-dependent plasma glycoprotein (PROZ)	0.676	0.03401
Hyaluronan-binding protein 2 (HABP2)	0.324	0.00029
		
**Metabolism**		
Sex hormone binding globulin (SHBG) [68]	1.381	2.30E-07
Insulin-like growth factor binding protein 1 (IGFBP1) [18]	1.318	3.82E-05
Insulin-like growth factor binding protein 4 (IGFBP4) [18]	0.773	8.61E-06
Apolipoprotein A-II (APOA2) [57]	0.379	4.06E-06
Vitamin D binding protein (GC) [59]	0.298	5.82E-06
Apolipoprotein D (APOD) [57]	-0.396	0.00133
Insulin-like growth factor binding protein 6 (IGFBP6)	0.303	0.00225
Insulin-like growth factor (IGF1) [18]	-0.410	0.00366
Proprotein convertase subtilisin kexin 9 (PCSK9)	0.385	0.02486
Serpin peptidase inhibitor, clade A, member 6 (SERPINA6)	0.377	0.02446
		
**Osteogenesis**		
Fetuin B (FETUB)	0.748	2.81E-07
Macrophage stimulating protein 1 (MST1)	0.546	0.00154
Collagen type 1, alpha 1 (COL1A1)	-0.494	0.00023
Tolloid-like protein 1, bone morphogenetic protein 1 (TLL1)	-1.150	0.0467
Neurogenic locus notch homolog protein 2 (NOTCH2)	-0.289	0.01946
Neurogenic locus notch homolog protein 3 (NOTCH3)	-0.622	0.02133
Fetuin A (AHSG) [59]	0.281	1.16E-06
		
**Cell growth**		
Inhibin, beta E (INHBE)	0.472	0.01866
Follistatin-like 3 (FSTL3)	-0.353	0.02042
Transforming growth factor-beta-induced protein ig-h3 (TGFBI)	0.322	0.0036
		
**Complement and immune response**		
Serpin peptidase inhibitor, clade G, member 1 (SERPING1)	0.551	0.01216
Complement C2 (C2)	0.333	0.00215
Complement factor H-related protein 5 (CFHL5)	0.294	6.72E-05
Complement factor B (BF)	0.271	1.06E-06
Pantetheinase (VNN1)	0.564	0.00079
Leucine-rich alpha-2-glycoprotein (LRG1)	0.539	0.00031
Neutrophil defensin 1 (DEFA1)	0.303	0.00683
Mannose-binding protein C (MBL2)	-0.300	0.00094
TRAF-type zinc finger domain-containing protein 1 (TRAFD1)	-3.863	0.00762
Lactotransferrin (LTF) [69]	0.285	0.04264
Trefoil factor 3 (TFF3)	1.936	0.00019
		
**Vessel morphogenesis**		
Autotaxin (ENPP2)	0.581	0.00395
Vasorin (SLITL2)	-0.383	0.01997
Transgelin 2 (TAGLN2)	-0.542	0.01725
Endothelial differentiation G-protein coupled receptor 3 (EDG3)	-2.998	0.00033
Cardiomyopathy associated protein 5 (CMYA5)	-4.1374	0.01562
		
**Other**		
Angiotensinogen (AGT) [15,16]	1.148	7.16E-10
Cathepsin S (CTSS)	0.588	0.04665
Galectin-3-binding protein (LGALS3BP)	0.416	0.00214
Galectin 1 (LGALS1)	0.305	0.02924
E3 ubiquitin-protein ligase UBR1 (UBR1)	-0.422	0.00511
Tropomyosin alpha-4 chain (TPM4)	-1.258	0.0269
DNA helicase B (HELB)	-1.862	0.02157
Putative Polycomb group protein ASXL1 (ASXL1)	-2.658	0.02290
Protein CREG2 (CREG2)		
Protein RIC1 homolog (KIAA1432)	-4.153	0.00155
Protein FAM59B (FAM59B)	-2.755	0.00119
KH homology domain-containing protein 1 (C6orf148)	-3.060	0.00116
Alpha-1B-glycoprotein (A1BG)	0.331	1.82E-06
Disks large homolog 2 (DLG2)	1.749	0.04913

Proteins with prior associations with ET are indicated with numbered references.

A critical step in estrogen effect on gene expression is recognition of the estrogen response elements (EREs) via estrogen receptors. For the differentially expressed proteins, we checked for the presence of conserved (between mouse and human) EREs in their corresponding genes. The sequence match was performed against a publicly available ERE database [36]. Four proteins - AGT, galectin-1 (LGALS1), LTF, and trefoil factor 3 (TFF3) - found to be significantly elevated with CEE in our study, had conserved EREs upstream of the coding region. None of the down-regulated proteins had conserved EREs upstream of the coding regions of their genes. However, one down-regulated protein (matrix metalloproteinase 2 (MMP2)) had an ERE in the downstream region of its corresponding gene.

### Validation of a set of proteins up-regulated with ET

**Figure 3:**
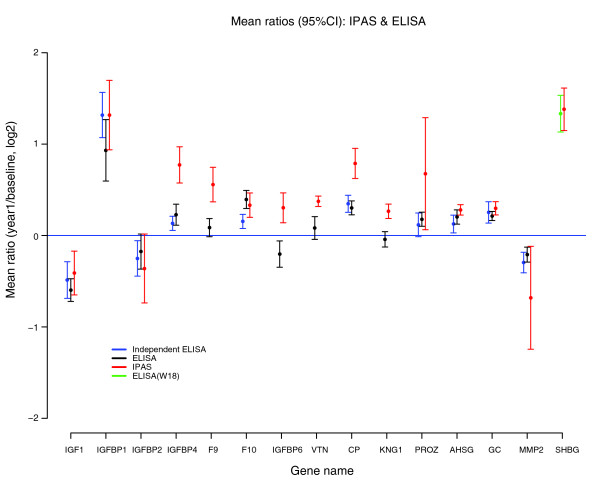
Mean ratios (95% confidence intervals (CI)) for MS-based (IPAS, shown in red) and ELISA-based quantification (shown in black for the same set of 50 sera analyzed by MS and in blue for the independent set of 50 sera). SHBG ELISA data were based on a separate independent set of sera.

**Figure 4:**
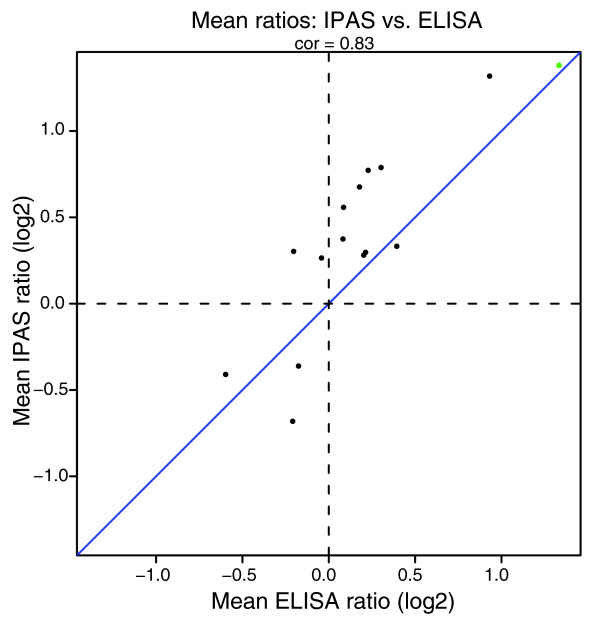
Comparison of mean ratios (1 year ET/baseline) by IPAS MS and by ELISA.

**Figure 5:**
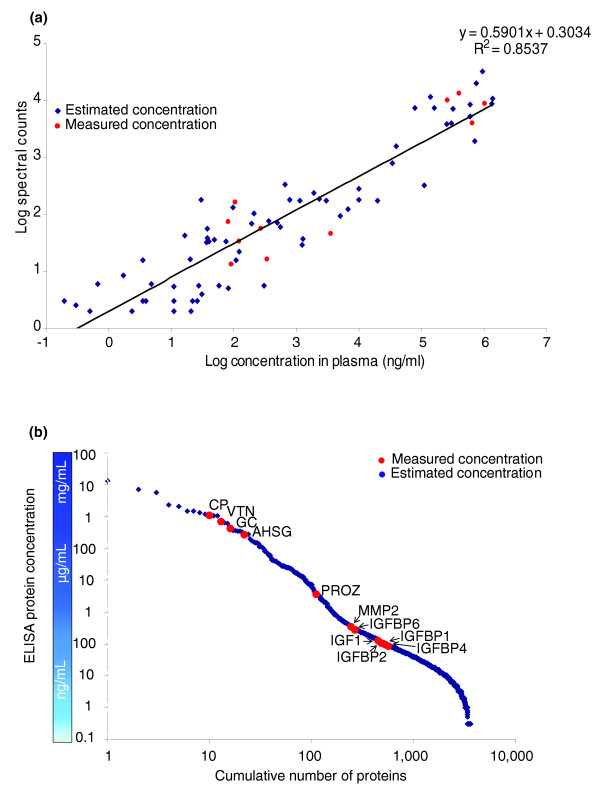
Dynamic range of IPAS MS pointing to proteins validated by ELISA. **(a) **Correlation between spectral counts (number of tandem mass spectra (MS2) acquired per protein) and estimated/measured serum concentrations. **(b) **Cumulative protein identifications are plotted versus ELISA protein concentration determined by ELISA measurments (red) and estimated concentration (blue) as determined by spectral counts.

We sought to validate proteomic data by ELISA analysis of individual non-pooled sera from the same subjects in the study. Proteins were selected for assay among the set of 64 proteins meeting statistical criteria for change following CEE, based on availability of a pair of antibodies with the requisite specificity for ELISA-based validation. Thus, assays were available for IGF1, IGFBP4, IGFBP1, IGFBP6, F9, F10, AHSG, vitronectin (VTN), GC, CP, MMP2, kininogen (KNG1), and PROZ. In addition, IGFBP2 was tested as a negative control. SHBG was separately analyzed in a set of 50 women in the trial, who had similar characteristics to those in the training set. High-density lipoprotein and low-density lipoprotein were previously tested and, therefore, were not subjected to additional validation in our study [6]. Figure 3 presents the data at baseline and 1 year for each protein. The correlation between IPAS proteomic log-ratios and ELISA log-ratios was strong (correlation = 0.83 without SHBG and 0.86 with SHGB; Figure 4). We obtained a correlation of 0.85 between spectral counts (number of tandem mass spectrometry (MS2) events/protein) and the known serum concentrations of more than 80 proteins (Figure 5a). The measured abundance range of the proteins subjected to ELISA (Figure 5b) is indicative of the depth of proteomic analysis in this study, which was achieved through extensive fractionation of intact proteins and reliance on high-resolution MS, spanning seven logs of protein abundance. However, low abundance proteins are somewhat under-sampled, given that proteins quantified in more than two proteomic experiments only spanned some four logs of protein abundance.

### Validation studies in an independent set of sera

We further analyzed an additional, independent validation set of 50 non-overlapping randomly selected women, who adhered to CEE over the first year of randomization in the CEE trial, for IGF1, IGFBP4, IGFBP1, F10, AHSG, GC, CP, MMP2, and PROZ and for IGFBP2 as a negative control (Figure 2). The correlation between ELISA results for the training set and the independent test set was 95%, and between the independent set tested by ELISA and the training set tested by IPAS it was 87%. Elevated concentrations at 1 year from randomization compared to baseline were observed in these independent samples for all ten proteins studied.

## Discussion

The objective of this proteomic study was to determine whether an in-depth, unbiased, quantitative analysis of serum proteins in a clinical trial setting would uncover changes that are relevant to the objectives of the clinical trial, thereby supporting the utility of comprehensive profiling of the serum proteome for clinical investigations. The choice of clinical trial for this study, namely the WHI CEE randomized controlled trial, is significant from the point of view of health effects observed, which include an adverse effect on stroke and venous thromboembolism and a reduction of hip fractures. Additionally, given that some findings have been published with respect to the effect of CEE on a selected set of serum proteins, there was an opportunity to assess concordance of proteomics-derived data with previously observed findings and to assess the potential of proteomics to uncover novel protein changes related to oral ET. We used acrylamide isotopic labeling of cysteine residues to obtain quantitative data for changes in serum proteins between baseline and 1 year after CEE for 50 subjects. This labeling approach is chemically very efficient as shown by the lack of unlabeled cysteines in searched mass spectra [19]. It would be expected given the number of proteins quantified that approximately 31 proteins would satisfy a nominal *P *< 0.05 selection criterion under a global null hypothesis. The number of quantified proteins that reached this threshold of statistical significance was 116, which represented a sizeable fraction (19%) of the proteins with quantitative measures and is indicative of a substantial effect of CEE on the serum proteome, based on a systematic, unbiased analysis.

It was of interest to determine the contribution of EREs to upregulation of protein levels with oral ET. The genes for four up-regulated proteins contained conserved EREs. *LTF *is a well known estrogen-regulated gene [37-40]. As with all classical estrogen target genes, the human and mouse orthologs of LTF both contain an ERE at a similar location in their promoter region, and are most sensitive to estrogen stimulation in the reproductive organs [39,40]. The human *AGT *gene includes an ERE close to the TATA box in its promoter region, which may be responsible for its increased transactivation by estrogen [41]. The *TFF3 *gene, which plays a role in mucosal protection and repair in the gastrointestinal tract, is known to be induced by estrogen [42], and it is over-expressed in several types of cancer [43]. Elevated serum levels of TFF3 have been reported in inflammatory bowel disease [44] and ulceration of the upper gastrointestinal tract [45]. LGALS1 was shown to be induced by estrogen [46]. One down-regulated protein (MMP2) had an ERE in the downstream region of the gene. In one study, estrogen was shown to increase MMP2 activity and protein expression in human granulosa lutein cells [47]. In another study, treatment with low dose estrogens increased MMP2 expression and activity. However, estrogens at a similar level as in the case of women receiving hormone replacement therapy failed to up-regulate MMP2 expression and activity [48]. The human MMP2 promoter contains several potential *cis*-acting regulatory elements, including cAMP response element-binding protein (CREB), AP-1, PEA3, C/EBP, P53, Est-1, AP-2, and Sp1 binding sites [49,50]. This may suggest that regulation of *MMP2 *gene expression is not primarily through the classic ERE-mediated pathway [1]. Given that most up-regulated proteins with oral ET do not display a conserved ERE in their corresponding genes, it would follow that their upregulation is likely through other mechanisms.

Up-regulated serum levels were observed for as many as nine proteins that play a role in coagulation (PLG, F9, F10, factor XII (F12), KNG1, PROZ, SERPING1 (serpin peptidase inhibitor, clade G, member 1), VTN, and FGG (fibrinogen gamma chain)), which may be relevant to the increased risk of venous thromboembolism and stroke with CEE. Of these, PLG [51], FGG, F12, and high molecular weight KNG1 [52] have been reported to increase with ET. The last three of these are components of the plasma kallikrein-kinin system, which mediates changes in coagulation, inflammation and blood pressure, all of which may contribute to atherothrombosis. Increased levels of PROZ, F9, F10, VTN, FGG, and platelet basic protein (PPBP) are novel findings. PROZ is structurally related to F9 and F10, and serves as a cofactor for the inactivation of activated F10. A case-control study found a strong, independent relationship between elevated blood levels of PROZ and ischemic stroke during the acute phase [53]. Thus, our results are consistent with the notion that PROZ might be an important factor in the pathogenesis of ischemic stroke in postmenopausal women receiving CEE. Vascular smooth muscle cells constitutively elaborate the zymogen form of MMP2. When activated, MMP2 promotes vascular lesion development [54].

Our data indicate that IGF1/IGFBP levels were significantly changed after 1 year of CEE, in accordance with data from a small randomized study of 35 healthy postmenopausal women in which circulating IGF1 levels were significantly reduced by CEE and plasma concentrations of IGFBP1 and IGFBP4 increased from baseline [18]. We confirmed in this larger study that CEE increased the IGFBP1 and IGFBP4 serum levels from baseline to 1 year of ET and decreased IGF1.

We observed for the first time CEE related increased levels of proprotein convertase subtilisin kexin 9 (PCSK9), which regulates low-density lipoprotein receptor levels. Mutations in the *PCSK9 *gene have been associated with CHD risk [55,56]. Our data confirm previously reported high levels of APOA2, a major component of high-density lipoprotein, with CEE [57]. We also found that SERPINA6 (serpin peptidase inhibitor, clade A, member 6), the major transporter for glucorticoids and progestins in the blood, is elevated after CEE. It has been negatively correlated with insulin resistance and body mass index [58]. Conversely, increased blood levels of GC [59] with CEE are associated with obesity and insulin resistance [60]. Thus, through several pathways, estrogen appears to have effects on cardiovascular risk characteristics.

We found that several proteins from the inflammation, innate immunity and complement cascade were elevated after CEE, suggestive of a low grade inflammatory state, consistent with previously reported CEE-induced increases in C-reactive protein [14]. Some proteins implicated in cellular growth had increased levels with CEE (LTF, inhibin, beta E (INHBE), IGFBPs) whereas others were decreased (follistatin-like 3 (FSTL3), IGF1). Interestingly, we found changes in five proteins (AHSG, fetuin B (FETUB), macrophage stimulating protein 1 (MST1), collagen type 1, alpha 1 (COL1A), tolloid-like protein 1, bone morphogenetic protein 1 (TLL1)) directly implicated in osteogenesis and several others (IGF/IGFBPs, MMP2, NOTCH-1 and 3) that play a role in osteogenesis. These findings are of interest given the reduction in fractures with CEE.

AGT, a potent blood pressure vasoconstrictor, occurred at increased levels following CEE as previously observed [15,16]. Increases in levels of proteins from the plasma kallikrein-kinin system also suggest an impact of CEE on blood pressure regulation, although this has not been borne out in blood pressure measurements of women taking CEE.

Changes in levels of several proteins implicated in blood vessel morphogenesis and angiogenesis were observed. Autotaxin (ENPP2), an angiogenic factor and stimulant for cellular growth, was found to be increased whereas other proteins (transgelin 2 (TAGLN2), endothelial differentiation G-protein coupled receptor 3 (EDG3), cardiomyopathy associated protein 5 (CMYA5)) were decreased. MMP2, which promotes vascular lesion development [54], is decreased, as is SLITL2 (vasorin), which contributes to neointimal formation after arterial injury [61]. Changes in these proteins may have an effect on vasculature within 1 year of CEE.

Our proteomics study also confirmed that levels of lipoprotein APOA2, which is CHD protective, are up-regulated, while levels of APOD are down-regulated and apolipoprotein A (LPA) not changed, in accordance with previous findings from the WHI study [51]. The plasma kallikrein-kinin system has been implicated in cardiovascular disease in men, but activation of this system has not been specifically investigated in individuals at risk for CHD [62].

Reduction of hip fractures is a well known effect of CEE and, interestingly, we found that ossification was a major significant affected network. Changes in five proteins (AHSG, FETUB, MST1, COL1A, TLL1) directly implicated in osteogenesis were observed and several others (IGF/IGFBPs, MMP2, NOTCH-1) that play a role in osteogenesis exhibited altered levels with CEE.

To further support our proteomics findings, we measured by ELISA a subset of deregulated proteins using the same sera in our training set and in an additional validation set of 50 women. Our data showed a strong correlation between ELISA and MS results in both test and validation sets, reflecting reliability of MS and isotopic labeling for protein quantification. For the three proteins where ELISA measurements did not confirm the IPAS ratios, it is difficult to precisely determine the cause of the discrepancies. It is possible that different species are measured by ELISA versus IPAS (that is, different isoforms). Since the epitopes of the antibodies used in ELISAs are often not specified or ambiguous, it is difficult to conclusively determine if this is the case.

The findings presented here relate specifically to the effect on the serum proteome of orally administered postmenopausal ET. It is well know that the effect of estrogen depends on the route of administration [63,64]. For example, in one study, IGF-1 concentrations were found to decrease significantly with oral estrogen, whereas no significant change was observed with transdermal estrogen at 6 months [63]. Given the oral route of administration of estrogen in our study, it was of interest to determine the organ source of affected proteins. A search of gene expression data in SymAtlas [65] indicated that approximately half of the 62 proteins that were dysregulated with oral CEE in our study had the liver as their major organ source.

Protein changes after oral ET in postmenopausal women observed in this study indicate a substantial effect on coagulation and metabolic proteins that may explain the increased risk of venous thromboembolism and stroke and the reduced risk of fracture found in the WHI trial. Contributions of the route of administration of estrogen (oral versus transdermal) and dosage to effects on the serum proteome require further study, and our findings may not be directly relevant to parenteral routes of delivery or lower doses. We note that transdermal estrogen has not been linked to an increased risk of venous thromboembolism in a recent large meta-analysis [66].

## Conclusions

In-depth proteomic MS analysis of plasmas obtained from subjects in the WHI hormone replacement therapy trial uncovered 116 proteins (19%) that exhibited quantitative changes 1 year after CEE. Protein changes were related to processes that included coagulation, metabolism, osteogenesis, inflammation, and blood pressure maintenance. Findings for selected proteins were confirmed in the initial set of plasmas using ELISA, and further validated in an independent set of samples. This in-depth proteomic study has shown that a substantial fraction of the serum proteome is affected by CEE. The observed changes have relevance to findings from the clinical trial. This study points to the potential for proteomic investigations to provide a quantitative assessment of changes in the proteome that could elucidate effects of various interventions as part of clinical trials, and that form the basis of further investigations.

## Abbreviations

AGT: angiotensinogen; AHSG: fetuin A; APOA2: apolipoprotein A-II; APOD: apolipoprotein D; CEE: conjugated equine estrogens; CHD: coronary heart disease; COL1A: collagen type 1, alpha 1; CP: ceruloplasmin; EDG3: endothelial differentiation G-protein coupled receptor 3; ELISA: enzyme-linked immunosorbent assay; ERE: estrogen response element; ET: estrogen therapy; F9: coagulation factor IX; F10: coagulation factors X; F12: coagulation factor XII; FDR: false discovery rate; FGG: fibrinogen gamma chain; GC: vitamin D binding protein; IGF: insulin-like growth factor; IGFBP: insulin-like growth factor binding protein; INHBE: inhibin, beta E; IPAS: intact protein analysis system; KNG1: kininogen; LGALS1: galectin-1; LTF: lactotransferrin; MMP2: matrix metalloproteinase 2; MS: mass spectrometry; MST1: macrophage stimulating protein 1; PCSK9: proprotein convertase subtilisin kexin 9; PLG: plasminogen; PROZ: protein Z, vitamin K-dependent plasma glycoprotein; SERPING1: serpin peptidase inhibitor, clade G, member 1; SHBG: sex hormone binding globulin; TFF3: trefoil factor 3; TLL1: tolloid-like protein 1, bone morphogenetic protein 1; VTN: vitronectin; WHI: Women's Health Initiative.

## Competing interests

The authors declare that they have no competing interests.

## Authors' contributions

HK participated in the data acquisition, analysis, and interpretation. SP contributed to data analysis and interpretation, and carried out immunoassays. RP participated in the design of the study, statistical analysis, and data interpretation, and drafted the manuscript. AA performed the statistical analysis. VMF and SJP participated in the data acquisition and interpretation. QZ participated in the data analysis. HW performed data acquisition. MS and JK carried out immunoassays. JR, RJ, JH, RC, and JM contributed to the drafting of the manuscript. SH participated in the study design, data interpretation, and drafting of the manuscript.

## Additional data files

The following additional data are available with the online version of this paper. Additional data file 1 is an Excel document showing ratios (1 year CEE/baseline) of gene-level weighted proteins for each IPAS (log2scale), number of events identified for each unique gene and their *P*-values. Additional data file 2 is an Excel document showing weighted gene-level proteins quantified in two or more IPAS experiments with significant ratio 1 year ET/baseline (*P *< 0.05). Additional data file 3 is an Excel document showing proteins deregulated after 1 year ET with log-ratios >1.20 or <1/1.20.

## Supplementary Material

Additional data file 1 Ratios (1 year CEE/baseline) of gene-level weighted proteins for each IPAS (log2scale), number of events identified for each unique gene and their *P*-values. Click here for file

Additional data file 2 Weighted gene-level proteins quantified in two or more IPAS experiments with significant ratio 1 year ET/baseline (*P *< 0.05). Click here for file

Additional data file 3 Proteins deregulated after 1 year ET with log-ratios >1.20 or <1/1.20. Click here for file
